# Supplementary material for: Association between Body Roundness Index and Depression Among Middle-aged and Older Adults in Chinese Communities: An Empirical Analysis Based on CHARLS Data
Source: PLoS One. 2025 Mar 28;20(3):e0320139. doi: 10.1371/journal.pone.0320139 (PMC11952244; doi:10.1371/journal.pone.0320139)
Supplement: S2 File — The STROBE Statement can be obtained and assessed through the STROBE website [10] or the article by von Elm et al. [35]. For further details on its evaluation framework and criteria, refer to the comprehensive interpretation by Vandenbroucke et al [36]. The quality of reporting for each STROBE item is rated as “adequately reported,” “inadequately reported,” or “not applicable.” Five items (6a, 6b, 12d, 14c, and 15) are related to specific study designs. If an item is not applicable, it is rated as “not applicable” and is not included in the total number of items to be evaluated. (DOCX) [file pone.0320139.s002.docx]

**STROBE Statement—(Strengthening the Reporting of Observational Studies in Epidemiology) Checklist for Cross-sectional Studies**

**This checklist adheres to the STROBE 2022 guidelines to ensure comprehensive reporting of our population-based cross-sectional study investigating the association between body roundness index and depression prevalence in Chinese middle-aged and older adults. Each checklist item is addressed in the manuscript as follows:**

| **Section/item** | **Item No** | **Recommendation** | **Reported on Page Number/Line Number** | **Reported on Section/Paragraph** |
| --- | --- | --- | --- | --- |
| Title and abstract | 1 | (a) Indicate the study’s design with a commonly used term in the title or the abstract | Line 1 | Title |
| Title and abstract | 1 | (b) Provide in the abstract an informative and balanced summary of what was done and what was found | Line 27-42 | Abstract |
| Introduction | 2 | Explain the scientific background and rationale for the investigation being reported | Line 47-84 | 1. Introduction |
| Objectives | 3 | State specific objectives, including any prespecified hypotheses | Line 85-90 | 1. Introduction |
| Methods | 4 | Present key elements of study design early in the paper | Line109-110 | 2. Materials and Methods |
| Setting | 5 | Describe the setting, locations, and relevant dates, including periods of recruitment, exposure, follow-up, and data collection | Line93-108 | 2.1 Data Source |
| Participants | 6 | (a) Cohort study—Give the eligibility criteria, and the sources and methods of selection of participants. Describe methods of follow-up | Line 109-121 | 2.1 Data Source |
| Participants | 6 | (b) Cohort study—For matched studies, give matching criteria and number of exposed and unexposed | Not applicable | N/A |
| Variables | 7 | Clearly define all outcomes, exposures, predictors, potential confounders, and effect modifiers. Give diagnostic criteria, if applicable | Line 124-133 | 2.2 Measurement of Depression |
| Data sources/ measurement | 8 | For each variable of interest, give sources of data and details of methods of assessment (measurement). Describe comparability of assessment methods if there is more than one group | Line 136-142 | 2.3 Measurement of BRI |
| Bias | 9 | Describe any efforts to address potential sources of bias | Line 162-165 | 2.5 Statistical Analysis |
| Study size | 10 | Explain how the study size was arrived at | Line 100-104 | 2.5 Statistical Analysis |
| Quantitative variables | 11 | Explain how quantitative variables were handled in the analyses. If applicable, describe which groupings were chosen and why | Line 155-159 | 2.5 Statistical Analysis |
| Statistical methods | 12 | (a) Describe all statistical methods, including those used to control for confounding | Line 160-169 | 2.5 Statistical Analysis |
| Statistical methods | 12 | (b) Describe any methods used to examine subgroups and interactions | Line 170-175 | 3.4 Subgroup Analysis |
| Statistical methods | 12 | (c) Explain how missing data were addressed | Line 119-121 | 2.1 Data Source |
| Statistical methods | 12 | (d) Cohort study—If applicable, explain how loss to follow-up was addressed | Not applicable | N/A |
| Statistical methods | 12 | (e) Describe any sensitivity analyses |  | N/A |
| Results | 13 | (a) Report numbers of individuals at each stage of study—eg numbers potentially eligible, examined for eligibility, confirmed eligible, included in the study, completing follow-up, and analysed | Line 177-183 | 3.1 Baseline Characteristics |
| Results | 13 | (b) Give reasons for non-participation at each stage |  | NA |
| Results | 13 | (c) Consider use of a flow diagram |  | Figure 1 |
| Results | 14 | (a) Give characteristics of study participants (eg demographic, clinical, social) and information on exposures and potential confounders | Line 184-193 | 3.1 Baseline Characteristics |
| Results | 14 | (b) Indicate number of participants with missing data for each variable of interest | Line 100-104 |  |
| Results | 14 | (c) Cohort study—Summarise follow-up time (eg, average and total amount) | Line 100-104 | 2.1 Data Source |
| Results | 15 | Cohort study—Report numbers of outcome events or summary measures over time |  |  |
| Results | 16 | (a) Give unadjusted estimates and, if applicable, confounder-adjusted estimates and their precision (eg, 95% confidence interval). Make clear which confounders were adjusted for and why they were included | Line 199-206 | 3.2 Association between BRI and the Risk of Depression |
| Results | 16 | (b) Report category boundaries when continuous variables were categorized | Line 179-181 | 3.1 Baseline Characteristics |
| Results | 16 | (c) If relevant, consider translating estimates of relative risk into absolute risk for a meaningful time period | Line 199-206 | 3.2 Association between BRI and the Risk of Depression |
| Results | 17 | Report other analyses done—eg analyses of subgroups and interactions, and sensitivity analyses | Line 243-248 | 3.4 Subgroup Analysis |
| Discussion | 18 | Summarise key results with reference to study objectives | Line 252-259 | 4. Discussion |
| Discussion | 19 | Discuss limitations of the study, taking into account sources of potential bias or imprecision. Discuss both direction and magnitude of any potential bias | Line350-362 | 5. Conclusion |
| Discussion | 20 | Give a cautious overall interpretation of results considering objectives, limitations, multiplicity of analyses, results from similar studies, and other relevant evidence | Line 293-313 | 5. Conclusion |
| Discussion | 21 | Discuss the generalisability (external validity) of the study results | Line 317-324 | 5. Conclusion |
| Other information | 22 | Give the source of funding and the role of the funders for the present study and, if applicable, for the original study on which the present article is based |  |  |

*Give information separately for cases and controls in case-control studies and, if applicable, for exposed and unexposed groups in cohort and cross-sectional studies.

**Note:** An Explanation and Elaboration article discusses each checklist item and gives methodological background and published examples of transparent reporting. The STROBE checklist is best used in conjunction with this article (freely available on the Web sites of PLoS Medicine at http://www.plosmedicine.org/, Annals of Internal Medicine at http://www.annals.org/, and Epidemiology at http://www.epidem.com/). Information on the STROBE Initiative is available at www.strobe-statement.org.
