# Supplementary material for: Association between Body Roundness Index and Depression Among Middle-aged and Older Adults in Chinese Communities: An Empirical Analysis Based on CHARLS Data
Source: PLoS One. 2025 Mar 28;20(3):e0320139. doi: 10.1371/journal.pone.0320139 (PMC11952244; doi:10.1371/journal.pone.0320139)
Supplement: Attachment — Submitted filename: PONE-D-24-55220.pdf [file pone.0320139.s003.pdf]

Review Comments

Congratulations to the authors and the team on completing this manuscript. This study addresses a significant public health issue by exploring the relationship between Body Roundness Index (BRI) and depression risk among older Chinese adults. The use of robust statistical methods and a large dataset enhances the credibility of the findings. However, there are areas where further refinement would improve the clarity, rigor, and impact of the manuscript. Below are detailed comments and suggestions for each section.

General Comments

Abbreviations: While many abbreviations (e.g., BMI, BRI, CHARLS) may be familiar to readers, it is recommended to present the full term followed by the abbreviation upon first use. Please ensure consistency throughout the manuscript.

Language and Grammar: Several minor spelling and grammatical errors need attention, such as "behavio" in line 41, an extra ")" in line 63, and "Educational leve" in the tables. Line 136 ("were included?") and other grammatical issues also require correction. A thorough proofreading and professional English editing are strongly recommended. Please include an English editing certificate upon resubmission.

Introduction

The introduction is well-organized and provides a comprehensive overview of the topic, but it could be more focused and streamlined to highlight the core research question better.

Suggestions for Improvement

1. Focus on the Core Theme: The connection between BRI and depression, the central research question, is introduced too late in the introduction. Consider reorganizing the content to bring this focus forward.
2. Streamline Discussion on BMI: The section discussing BMI's limitations is overly detailed and could be condensed to maintain narrative flow.
3. Strengthen Transitions: Improve transitions between sections to make the progression from depression to BMI, and then to BRI, more seamless.

4. Highlight Research Significance: Clearly articulate why this study focuses on older Chinese adults and how it addresses gaps in the existing literature.

Proposed Structure for Rewriting

1. First Paragraph: Briefly introduce depression and its association with obesity, emphasizing the public health relevance.
2. Second Paragraph: Discuss BMI as a commonly used but limited measure of obesity, highlighting the need for alternative indicators like BRI.
3. Third Paragraph: Introduce BRI, its advantages over BMI, and its potential relationship with depression. Conclude by stating the research objectives and hypotheses.

Materials and Methods

The methods section is well-structured and provides detailed information about the dataset and statistical approaches. However, some clarifications and additional details would improve transparency.

Suggestions for Improvement

1. Data Source and Sample Selection: Clarify why Wave 3 and Wave 5 of the CHARLS dataset were chosen and address any potential selection bias.
2. Line 102: Include the CHARLS website URL as a reference.
3. Depression Measurement: Specify whether the CESD-10 scale used was the English or Chinese version. If the Chinese version was used, cite validation studies ensuring its reliability and validity. If none exist, mention this as a limitation and cite the original CESD-10 study.
4. Anthropometric Measurements: Indicate whether height, weight, and waist circumference were self-reported or measured by professionals. If measured, provide details about the equipment or personnel involved.
5. Clarify Gender and Sex: Use "gender" and "sex" appropriately throughout the manuscript, as these terms are not interchangeable in academic contexts.
6. Wave Consistency: Line 147: Confirm whether the 2015 baseline data correspond to Wave 1 or Wave 3, as the CHARLS website indicates Wave 1 refers to 2011. Clarify this discrepancy.
7. Explain the rationale for using Wave 3 BRI to predict Wave 5 depression risk without

considering Wave 5 BRI. Discuss whether changes in BRI between waves might influence the results.

8. Rewrite the sentence: ”Statistical analyses were conducted using R software (version X.X.X).”

Results

The results are presented clearly, with appropriate statistical models. However, there are areas where additional context and interpretation are needed to enhance understanding.

Suggestions for Improvement

1. Annotations in Tables and Figures: Ensure tables and figures can be understood independently. Provide full terms for abbreviations in table footnotes and include explanations of clinical or practical significance for the results (e.g., OR values and 95% CIs in Table 2). Clarify unclear elements, such as "P P for overall" in Figure 2.
2. Nonlinear Relationships: In Figure 2, annotate the key threshold (e.g., 4.29) and explain its significance for interpreting BRI’s impact on depression risk.
3. Model Fit: Include model fit indices (e.g., AIC, BIC) to justify and confirm the chosen models are optimal.
4. Dynamic Changes in BRI: Address whether changes in BRI between Wave 3 and Wave 5 influence depression risk. If fluctuations exist, discuss their potential implications.

Discussion

The discussion provides valuable insights into potential mechanisms linking BRI to depression but could benefit from additional depth and broader contextualization.

Suggestions for Improvement

1. Causal Inference: It would be beneficial to contextualize the limitations of causal inference further. Highlight the limitations of cross-sectional data and recommend more rigorous longitudinal or interventional studies to validate the findings. Consider the bidirectional relationship between BRI and depression, where depression might also contribute to obesity. Discuss how this bi-directionality could influence interpretation.

2. International Context: Compare findings with similar studies in other populations or regions to emphasize this study's novelty and broader relevance.
3. Mechanistic Hypotheses: Expand on the proposed mechanisms (e.g., inflammation, HPA axis dysregulation, gut microbiota) with more recent literature. Explain why these mechanisms are particularly relevant to middle-aged and older Chinese adults.
4. Practical Applications: Discuss how BRI could be integrated into clinical practice or public health policies, including the feasibility of using the 4.29 threshold in depression screening programs.

Final Remarks

Congratulations to the authors and the team for studying such an important topic. The use of an obesity index (BRI) in the context of depression risk is relevant. Addressing the above suggestions will help refine the manuscript, enhance its clarity and rigor, and increase its contribution to the field in China. I encourage the authors to continue this valuable research and look forward to seeing its further development.
