# Supplementary material for: Association between Body Roundness Index and Depression Among Middle-aged and Older Adults in Chinese Communities: An Empirical Analysis Based on CHARLS Data
Source: PLoS One. 2025 Mar 28;20(3):e0320139. doi: 10.1371/journal.pone.0320139 (PMC11952244; doi:10.1371/journal.pone.0320139)
Supplement: Attachment — Submitted filename: Response to Reviewers.docx [file pone.0320139.s004.docx]

**Dear Editor and reviewers,**

We sincerely appreciate the time you dedicated to providing constructive feedback on our manuscript. Your insightful comments have significantly enhanced its quality. In response to your suggestions, we have made the necessary changes, which are highlighted in red in the manuscript and supplementary materials. Below, we present a point-by-point response to the comments from both the editor and the reviewers. Thank you once again for your valuable input.

**GENERAL COMMENT**

**1.** Abbreviations: While many abbreviations (e.g.BMI, BRI, CHARLS) may be familiar to readers, it is recommended to present the full term followed by the abbreviation upon first use. Please ensure consistency throughout the manuscript.

**Author Response:** We greatly appreciate your valuable feedback on our manuscript. Concerning the abbreviation standardization issue you raised, we have comprehensively revised the terms such as BMI, BRI, and CHARLS according to the "full name - abbreviation" format principle. This ensures that all abbreviations are accompanied by their complete spellings upon their first appearance and carefully checked the consistency throughout the text to comply with standardized expression.

**2.** Language and Grammar: Several minor spelling and grammatical errors need attention, such as "behavio" in line 41, an extra ")" in line 63, and "Educational leve" in the tables. Line 136 ("were included?") and other grammatical issues also require correction. A thorough proofreading and professional English editing are strongly recommended. Please include an English editing certificate upon resubmission.

**Author Response:** We greatly appreciate your valuable feedback on our manuscript.

Regarding language and grammar: We have carefully proofread the entire manuscript based on your feedback and corrected all the specified spelling and grammatical errors, including the word “behavio” in line 41, the extra parentheses in line 63, and the “educational term” in the tables, as well as the grammatical issues mentioned in line 136 and other similar instances. To further enhance the language quality, we have engaged a professional English editing service and have attached the editing certificate. All revised content is highlighted in red.

**REVIEWER 1 EVALUATION**

**1.** The only comment that I have is regarding the tool used. The authors didn't specify what language the survey was presented in and, if it was translated, what methods were used to test the validity of the tool after the translation. There are a few typos that should be checked.

**Author Response:** We sincerely appreciate the valuable comments on our manuscript, particularly your attention to detail and the time spent on the review. Regarding the assessment tool for depression that you mentioned, the CES-D-10 is a brief self - rating scale for depressive symptoms, primarily used to screen for depressive symptoms in various populations, including the elderly. The Chinese version adopted in the CHARLS database has been proven to have good reliability and validity. We have also re - examined the reliability and validity data of the original CESD-10S scale and related literature has been cited in this paper. (please refer to line 119-130 on page 6 of the revised manuscript).

Regarding spelling errors, we have carefully reviewed and corrected all the errors identified throughout the manuscript and conducted additional proofreading to minimize the possibility of any residual mistakes. Moreover, we have invited a native English speaker to polish our article.

**REVIEWER ２ EVALUATION**

**1.** Suggestions for Improvement

**1.1** Focus on the Core Theme: The connection between BRI and depression, the central research question, is introduced too late in the introduction. Consider reorganizing the content to bring this focus forward.

**Author Response:** Thank you for your suggestions. According to your suggestion，We have revised the background introduction, introducing the correlation between BRI and depression earlier to highlight the core research question more effectively and focus on the elaboration of their relationship.

**1.2** Streamline Discussion on BMI: The section discussing BMI’s limitations is overly detailed and could be condensed to maintain narrative flow.

**Author Response:** Thank you for your suggestions. We streamlined the discussion on the limitations of BMI, retained core points and removed redundant information. Thanks again for helping to enhance the clarity and fluency of the manuscript! The revisions have been highlighted in the document, and I look forward to your further feedback.

**1.3** Strengthen Transitions: Improve transitions between sections to make the progression from depression to BMI, and then to BRI, more seamless.

**Author Response:** Thank you for your valuable comments on the logical coherence of our paper. We fully recognize the significant impact of paragraph transitions on readability. During the revision process, we systematically reviewed the logical progression from “depression to BMI to BRI,” and added transitional sentences and explanatory phrases to enhance the smoothness of our arguments.

**1.4** Highlight Research Significance: Clearly articulate why this study focuses on older Chinese adults and how it addresses gaps in the existing literature.

**Author Response:** Thank you for your crucial suggestions on the positioning of the study's innovation. We fully agree with the academic value of clarifying the uniqueness of the study population. In the last paragraph of the Introduction, we have emphasized the reasons for focusing on middle-aged and older adults in China. The population of older adults in China is expanding at a rapid pace, and this demographic is particularly vulnerable to depression, which can exert a profound impact on their physical health, cognitive function, and overall quality of life. Individuals aged 60 and above in China face a higher risk of depression, with 33.1% exhibiting depressive symptoms and 5.3% suffering from major depressive disorder. By examining the association between BRI and depressive symptoms, we aim to identify potential modifiable risk factors that could be targeted in prevention and treatment strategies.

However, most existing studies are based on Western populations, and their conclusions are difficult to directly apply to the Chinese elderly population, which is influenced by unique lifestyles, sociocultural contexts (such as family structure, social support systems, and cultural values). Therefore, our study fills this gap by conducting research on this specific population.

**2.** Proposed Structure for Rewriting

**2.1** First Paragraph: Briefly introduce depression and its association with obesity, emphasizing the public health relevance.

**Author Response:** Thank you for your suggestions. We have revised the content according to your suggestions, strengthened the introduction of depression and its relationship with obesity, and emphasized its relevance to public health. (Please refer to lines 47-62 on page 3).

For your convenience, the revised content is also provided here:

Depression and obesity are global health challenges that impact public health. The main features of depression include persistent mood disturbances, often accompanied by cognitive impairment, and deterioration of physical and social functioning. Severe cases may lead to suicide ^[1]^. Depression affects patients' quality of life, social functioning, and creates a large economic burden for families and society ^[2]^. The World Health Organization reports that about 350 million people global suffer from depression, with an increasing incidence rate yearly ^[3]^. In China, about 33.1% of adults aged 60 and above show depression symptoms, with a prevalence of major depressive disorder reaching 5.3% ^[4]^. Elderly people are more likely to suffer from depression due to declining physiological functions, social role changes, and increased chronic disease rates ^[5]^. The causes of depression involve genetics, neurobiology, and social influences ^[6, 7]^. Additionally, depression exhibits high heterogeneity and uncertainty in symptoms, progression, prognosis, and treatment response, making treatment and management challenging ^[8]^. Therefore, strengthening early identification and prevention of depression is crucial for effectively curbing the disease progression.

**2.2** Second Paragraph Discuss BMI as a commonly used but limited measure of obesity, highlighting the need for alternative indicators like BRI.

Author Response: Thank you for your suggestions. We have revised the content according to your suggestions, streamlined the introduction of obesity, and enhanced the discussion on the limitations of BMI, highlighting the necessity of exploring alternative indicators such as BRI to more comprehensively assess obesity and its related health risks. (Please refer to lines 63-67 on page 3 and lines 68-76 on page4 ).

For your convenience, the revised content is also provided here:

Evidence shows that obesity is an important risk factor for depression. Obese individuals have a 1.29 to 1.69 times higher risk of depression than those with normal weight ^[9]^. Traditionally, Body Mass Index (BMI) and Waist Circumference (WC) are commonly used to assess obesity, but both WC and BMI have their limitations in evaluating obesity.BMI cannot distinguish fat distribution from muscle mass, and WC as an indicator of obesity assessment also has its inherent limitations ^[10]^. In addition, There is a complex nonlinear association between obesity and mortality, called the "obesity paradox" ^[11]^, which challenges the application of traditional obesity indicators in health risk assessment. Studies on the association between obesity and depression show complexity, with some suggesting obesity increases depression risk ^[12]^ and others suggesting a protective effect ^[13]^. This inconsistency highlights the need for a more detailed evaluation of traditional indicators like BMI and calls for the exploration of more accurate methods for measuring obesity phenotypes.

**2.3** Third Paragraph Introduce BRI, its advantages over BMI, and its potential relationship with depression. Conclude by stating the research objectives and hypotheses.

**Author Response:** Thank you for your suggestions. We have revised the relevant sections according to your suggestions, providing a clearer introduction to the BRI and elaborating on its advantages over the BMI and its potential link to depression. We have highlighted the strengths of BRI in capturing body fat distribution, especially central obesity. Additionally, we have summarized the preliminary studies supporting the potential association between BRI and depression and clearly stated the objective of our study: to investigate whether BRI can more effectively predict depression risk than BMI. (Please refer to lines 77-90 on page 4).

For your convenience, the revised content is also provided here:

The body roundness index (BRI) is a new measure of obesity that combines WC and height, providing a more accurate reflection of abdominal fat accumulation and individual body characteristics ^[14]^. Compared to BMI, BRI can more accurately assess the severity of obesity ^[15]^. Studies have demonstrated that BRI can effectively forecast the risk of chronic conditions ^[16,17]^. A study based on the National Health and Nutrition Examination Survey (NHANES) revealed a positive correlation between BRI and depression in adults ^[18]^, but the applicability of this conclusion in middle-aged and older adults still requires further validation. Given the accelerated aging of China's population and the rising prevalence of depression among middle-aged and older adults, it is important to explore the connection between BRI and depressive symptoms in this demographic. Therefore, this research seeks to explore the relationship between BRI and depression using data from the China Health and Retirement Longitudinal Study (CHARLS), providing scientific evidence for the prevention and intervention of obesity and depression.

**3.** Materials and Methods

**3.1** The methods section is well-structured and provides detailed information about the dataset and statistical approaches. However, some clarifications and additional details would improve transparency. Data Source and Sample Selection: Clarify why Wave 3 and Wave 5 of the CHARLS dataset were chosen and address any potential selection bias.

**Author Response:** Thank you for your suggestions. When selecting the Wave 3 (2015) and the wave 5 (2020) of the CHARLS dataset to explore the association between the Body Roundness Index (BRI) and depressive symptoms among middle-aged and older Chinese adults, our consideration was primarily based on the long-term development characteristics of depressive symptoms. Depressive symptoms are often the result of a long-term process, shaped by the interaction of multiple factors over an extended period. Therefore, to thoroughly investigate this association, a dataset with a sufficient time span is necessary. The 5-year interval provides a valuable temporal window that allows us to capture the long-term and subtle relationships between depressive symptoms and BRI.

For the convenience of reviewers and readers, we have revised the relevant paragraph to read: “This study utilizes the CHARLS dataset, with the 2015 baseline data and the 2020 follow-up data for longitudinal analysis.”

**3.2** Line 102: Include the CHARLS website URL as a reference. https://charls.pku.edu.cn/

**Author Response:** Thank you for your suggestions. We have added the URL of the CHARLS website in the references.: https://charls.pku.edu.cn/(Please refer to lines 104 on page 5).

**3.3** Depression Measurement: Specify whether the CESD-10 scale used was the English or Chinese version. If the Chinese version was used, cite validation studies ensuring its reliability and validity. If none exist, mention this as a limitation and cite the original CESD-10 study.

**Author Response:** We sincerely appreciate the valuable comments on our manuscript. Regarding the assessment tool for depression that you mentioned, the CES-D-10 is a brief self - rating scale for depressive symptoms, primarily used to screen for depressive symptoms in various populations, including the elderly. The Chinese version adopted in the CHARLS database has been proven to have good reliability and validity. We have also re - examined the reliability and validity data of the original CESD-10S scale and its translated versions and cited relevant literature to substantiate this (please refer to lines 119 - 130 on page 6 ).

**3.4** Anthropometric Measurements: Indicate whether height, weight, and waist circumference were self-reported or measured by professionals. If measured, provide details about the equipment or personnel involved.

**Author Response:** Thank you for your suggestions. Regarding your query about the height and waist circumference measurement methods used in the CHARLS database, we have verified the procedures and provide the following clarification:

Height Measurement:

For height measurement, a wall-mounted stadiometer was employed. Participants stood barefoot with heels together, toes angled approximately 60 degrees, and back fully contacting the vertical board. The head was positioned in the Frankfort horizontal plane (aligned with the ear tragus and lower orbital rim). Trained technicians gently lowered the horizontal headplate until firm contact with the crown was achieved, with measurements recorded to the nearest 0.1 cm.

Waist Circumference Measurement:

Waist circumference was measured using a non-elastic tape. Participants maintained a relaxed standing posture with feet together and arms hanging naturally. The tape was positioned horizontally at the midpoint between the lowest rib margin and iliac crest (corresponding to the natural waistline), ensuring snug contact without compressing subcutaneous tissue. Measurements were taken at the end of normal expiration and recorded to the nearest 0.1 cm.

We hope this clarification adequately addresses your concerns and demonstrates our commitment to methodological transparency. Should additional details be required, we will provide supplementary documentation from the CHARLS operations manua (Please refer to lines 134 on page 6).

3.5 Clarify Gender and Sex: Use "gender" and "sex" appropriately throughout the manuscript, as these terms are not interchangeable in academic contexts.

**Author Response:** Thank you for your suggestions. We have carefully reviewed the manuscript and replaced “sex” with “gender” in the following sentences. We believe that these revisions clarify the use of “gender” and “sex” in our manuscript and align with academic conventions. We hope that our revised manuscript meets your standards.

3.6 Wave Consistency: Line 147: Confirm whether the 2015 baseline data correspond to Wave 1 or Wave 3, as the CHARLS website indicates Wave 1 refers to 2011. Clarify this discrepancy.

**Author Response:** Thank you for your suggestions. Thank you for pointing out the question in line 147. We confirm that the 2015 baseline data cited in the text corresponds to the third wave of the CHARLS survey. The first wave of CHARLS did indeed start in 2011, and the data from 2015 that we used comes from its third wave. The term “baseline data” mentioned in the text refers to the starting data for our study, not the initial wave of the entire CHARLS survey. To avoid any ambiguity, we will revise the wording in the text to clearly state that the data is sourced from the CHARLS 2015 (third wave) survey data. (Please refer to lines 151 on page 6).

**3.8** Explain the rationale for using Wave 3 BRI to predict Wave 5 depression risk without considering Wave 5 BRI. Discuss whether changes in BRI between waves might influence the results.

**Author Response:** Thank you for your attention to this issue. We chose Wave 3 as the baseline BRI for the following theoretical and research design considerations: The purpose of our study is to explore the predictive effect of baseline BRI on the risk of depression. As we mentioned earlier, depressive symptoms are a process that forms over a long period. To investigate this association, a sufficient temporal span is required. By setting the BRI in Wave 3 (2015) as the exposure variable, we can ensure that it precedes the depression symptom outcome in 2020, satisfying the temporal span and the causal relationship between them. If we were to consider including the BRI in Wave 5 (2020) at the same time, it might affect the rigorous logic of causality. Individuals with depressive symptoms may engage in emotional eating, which can also lead to changes in body weight and fat distribution, thereby affecting the BRI during the follow-up period. This bidirectional association would blur the independent effect of baseline BRI on the risk of depression, reducing the credibility of causal inference.

Regarding the second question, whether the change in BRI between Waves would affect the results. We fully agree with your viewpoint that changes in BRI can impact the risk of depression, and this is also a direction for our future research. In response to your question, we need to point out that if an individual's BRI undergoes significant changes between 2015 and 2020, it may be due to changes in metabolic status or psychological factors that have influenced the risk of depression. However, the focus of our study is on the independent predictive effect of baseline BRI, not the impact of dynamic changes. We acknowledge that, due to the limitations of our research design, this study did not delve into the association between the dynamic changes in BRI and the risk of depression. Future research will combine more Wave data from CHARLS to further reveal the relationship between changes in BRI and depressive symptoms.

**3.8** Rewrite the sentence: “Statistical analyses were conducted using R software (version X.X.X).”

**Author Response:** Thank you for your suggestions. We have revised the sentence according to your suggestion and it now reads: “Data cleaning and analysis were conducted using R software (version 4.4.1)”. (Please refer to lines 150 on page 6).

**4.** Results

**4.1** The results are presented clearly, with appropriate statistical models. However, there are areas where additional context and interpretation are needed to enhance understanding. Annotations in Tables and Figures: Ensure tables and figures can be understood independently. Provide full terms for abbreviations in table footnotes and include explanations of clinical or practical significance for the results (e.g., OR values and 95% CI in Table 2). Clarify unclear elements, such as "P P for overall" in Figure 2.

**Author Response:** Thank you for your suggestions. We have provided the full terms for all abbreviations in the footnotes of all tables. Additionally, we have added explanations of the clinical or practical significance of the results in the footnotes. For example, in Figure 2, we have clarified the meaning of “P for overall.” We have also reviewed all other figures to ensure that all elements are clear and understandable. (Please refer to lines 193-194 on page 12, lines 206-209 on page 12, lines210-212 on page 13 lines 229-230 on page 13, lines 513-522 on page 27).

For the specific issues you raised regarding Figure 2, for your convenience, we have placed the revised content here:

Figure 2. RCS diagram of the association between BRI and the risk of depression.

Note: Adjusted for the sex, age, place of residence, life satisfaction, sleep duration, social activities, pain, hypertension, diabetes, heart disease, stroke, cancer, chronic diseases, as well as smoking and alcohol consumption.

P for overall < 0.05 indicates the correlation between indicators and outcomes.

P for non-linear < 0.05 indicates that there is a nonlinear relationship between indicators and outcomes.

BRI is non-linearly associated with depressive risk. When BRI is less than 4.29, it has no significant impact on depressive risk; whereas when BRI exceeds 4.29, depressive risk significantly increases.

**4.2** Nonlinear Relationships: In Figure 2, annotate the key threshold (e.g., 4.29) and explain its significance for interpreting BRI’s impact on depression risk.

**Author Response:** Thank you for your suggestions. which is very important for enhancing the scientific nature of our research. In Figure 2, we found that the risk of depression shows a slow initial rise and then a rapid increase with the increase in BRI, with a turning point at BRI = 4.29. By performing logistic regression on the segmented models before and after the turning point, we confirmed the statistical significance of the threshold. The result analysis shows that when BRI < 4.29, P > 0.05, which is not statistically significant. However, when BRI > 4.29, the risk of depression increases significantly by 20% for every one-unit increase in BRI (OR = 1.20, 95% CI = 1.13-1.27, P < 0.0001). Through our study, the threshold of BRI = 4.29 can be used as a practical indicator for depression risk stratification in the middle-aged and elderly population. In clinical practice, doctors can calculate BRI through simple measurements of waist circumference and height, identify high-risk individuals (BRI > 4.29), and prioritize them for mental health assessment and lifestyle intervention (such as weight loss plans and anti-inflammatory diets). We have identified and explained the key threshold values in Figure 2. The pertinent information has been incorporated into the Results section of the article.

**4.3** Model Fit: Include model fit indices (e.g., AIC, BIC) to justify and confirm the chosen models are optimal.

**Author Response:** Thank you for reviewing our manuscript and pointing out these issues. We acknowledge the importance of model fit assessment. In our RCS analysis, we selected the optimal RCS model among 3 to 7 knots using the AIC criterion. The results indicated that the 4-knot model had the lowest AIC value (AIC = 925.748), significantly lower than the AIC values for other model knot configurations. This supports the rationale for choosing the 4-knot RCS model as the core analysis model. Following your suggestions, we have made the following revisions to the manuscript:

In the Methods section 1.5 Statistical Analysis, a new paragraph "Model selection is based on the AIC criterion, comparing the fit of different combinations of covariates, and ultimately determining the optimal model" has been added.

In the Results section 2.3 RCS analysis of the association between BRI and depression risk, a new paragraph "To explore the nonlinear relationship between BRI and depression risk, we conducted RCS analysis and selected the optimal RCS model among 3-7 nodes using the AIC criterion. The results showed that the 4-node model had the smallest AIC value and was used for subsequent analysis" has been added.

We acknowledge that not reporting the AIC in the initial draft was an oversight on our part. Thank you for pointing this out. The revised analysis now includes the model fit index AIC to ensure the transparency and scientific nature of model selection.

**4.4** Dynamic Changes in BRI: Address whether changes in BRI between Wave 3 and Wave 5 influence depression risk. If fluctuations exist, discuss their potential implications.

**Author Response:** Thank you for your comment. We fully agree with your perspective that the dynamic changes in BRI may impact the risk of depression. However, based on the study design and methodological considerations, we respond as follows:

The objective of this study was to investigate the independent predictive effect of baseline BRI (in 2015) on the risk of depression five years later (in 2020), rather than the association with dynamic changes. As we previously stated, by locking the baseline BRI as the exposure variable, we ensured that it preceded the depression - risk outcome temporally, in line with the principle of causal inference. If we had considered including BRI in 2020 as well, it might have created a bidirectional association, interfering with the independent interpretation of the baseline BRI effect and reducing the credibility of causal inference. Moreover, analyzing the impact of BRI dynamic changes on the risk of depression would require additional control of time - dependent confounding factors (such as changes in lifestyle and new - onset diseases between 2015 and 2020). However, such variables may be subject to measurement errors or may not have been fully captured. Therefore, the core objective of this study was to assess the long - term effect of baseline BRI, rather than its dynamic changes. Hence, we chose to simplify the model to enhance the robustness of the conclusions.

Future research can combine more waves of data from CHARLS and use methods such as trajectory models to analyze the association between BRI change patterns and the risk of depression. We believe that focusing on the independent effect of baseline BRI provides important evidence for the obesity - depression association, and the mechanism of dynamic changes will be a key focus of future research. The revised content has been highlighted, and we look forward to your further guidance.

**5.** Discussion

**5.1** The discussion provides valuable insights into potential mechanisms linking BRI to depression but could benefit from additional depth and broader contextualization. Causal Inference: It would be beneficial to contextualize the limitations of causal inference further. Highlight the limitations of cross-sectional data and recommend more rigorous longitudinal or interventional studies to validate the findings. Consider the bidirectional relationship between BRI and depression, where depression might also contribute to obesity. Discuss how this bi-directionality could influence interpretation.

**Author Response:** Thank you for your meticulous review and valuable feedback on this manuscript. We have revised the discussion section according to your suggestions, further deepening the exploration of mechanisms and adding an analysis of the limitations of causal inference and the bidirectional association. The specific revisions are as follows:

Regarding the limitations on causal inference (to be added to the limitations section):

We fully agree with your perspective on the limitations of causal inference. Despite the longitudinal design of this study, there may still be residual confounding factors, such as genetic predisposition, environmental exposure, or social support. These factors could potentially affect the accuracy of the results. Moreover, subclinical depressive symptoms within the cohort or depressive episodes during the follow-up period could still influence BRI through behavioral changes or metabolic dysregulation, thereby obscuring the independent effect of Wave 3 BRI.

Discussion on the bidirectional relationship:

Although this study focuses on the predictive effect of baseline BRI on depression risk, it is important to acknowledge the potential bidirectional association between the two. This study primarily examines the predictive effect of BRI on depression risk, but we have also supplemented the potential pathways by which depression could lead to obesity and discussed their impact on the interpretation of the results. The specific revisions are as follows:

In the discussion of the mechanisms section: Newly added

Obesity and depression may affect each other. On the one hand, obesity can raise depression risk through inflammation and HPA axis dysfunction ^[26]^. On the other hand, individuals with depression often experience appetite disorders (such as emotional eating), reduced physical activity, and metabolic side effects of antidepressants (such as weight gain), which could further exacerbate obesity. This bidirectional relationship suggests that the association between BRI and depressive symptoms may partially reflect the mutual reinforcement between the two conditions. Therefore, future research should employ methods such as cross-lagged panel models or mediation analysis to elucidate the temporal sequences and potential mediating mechanisms between obesity and depression.(Please refer to lines 304-313 on page 17 ).

**5.2** International Context: Compare findings with similar studies in other populations or regions to emphasize this study's novelty and broader relevance.

**Author Response:** Thank you to the reviewer for the important suggestions. Regarding the international context comparison of the study results, we have already compared our findings with similar studies in other populations or regions in the discussion section. We now further supplement and refine this as follows:

For example, a study involving 18,654 adults aged 20 and above demonstrated a notable positive link between BRI and the incidence of depression ^[18]^. In a model that comprehensively adjusted for age- and gender-related factors, for every unit increase in BRI, the prevalence of depression significantly rose by 8%. Another community survey based on NHANES showed that in the female group, the incidence rate of depression increased by 14% for each unit increase in BRI ^[26]^. However, current research on the relationship between BRI and depression primarily focuses on Western populations, with relatively little attention paid to the middle-aged and older adults in China. As one of the countries facing the most severe aging issues globally, the physical and mental health concerns of China's elderly population are in urgent need of attention. Our results indicate a dramatic positive correlation between BRI and depression risk among middle-aged and older adults in China. This finding is consistent with previous research conducted in American adults ^[18]^, further confirming the broad association between BRI as an obesity indicator and the risk of depression. By extending this association to the middle-aged and older adults in China, we provides a new perspective on understanding the relationship between BRI and depression in different ethnic and cultural backgrounds. In summary, this study not only fills the gap in research on the association between BRI and depression in middle-aged and older adults, but also provides valuable epidemiological evidence for the prevention and intervention of depression in China and potentially globally in response to the aging population. (Please refer to lines 258-273 on page 15 and lines 274-278 on page 16 ).

**5.3** Mechanistic Hypotheses: Expand on the proposed mechanisms (e.g., inflammation, HPA axis dysregulation, gut microbiota) with more recent literature. Explain why these mechanisms are particularly relevant to middle-aged and older Chinese adults.

**Author Response:** Thank you for your valuable suggestions. Regarding the hypothetical mechanisms linking obesity and depression, we have expanded and refined our discussion in the relevant sections (lines 276-295 on page 16 and lines 296-306 on page 17). We have not only reviewed the known potential biological mechanisms (such as inflammation, HPA axis dysregulation, and gut microbiota) but also supplemented our discussion with insights specific to the characteristics of the middle-aged and older Chinese population, based on the latest literature.

For your convenience, we have excerpted the revised content as follows:

Obesity can potentially escalate the risk of depression among middle-aged and older adults through several mechanisms. Firstly, with age, metabolic functions and antioxidant capacities typically diminish. The fat accumulation resulting from obesity further propels the production of reactive oxygen species (ROS). When ROS levels are excessive and the body's antioxidant capacity is insufficient, inflammatory signaling pathways can be activated, leading to damage of biomolecules and inducing cell apoptosis ^[27, 28]^. Moreover, the inflammatory responses triggered by obesity may intensify common neurodegenerative diseases in the elderly, such as Alzheimer's and Parkinson's diseases, thereby augmenting the risk of depression ^[29]^. Secondly, both depression and obesity share similar risk factors, one of which is an imbalance in gut microbiota. The gut microbiota can communicate with the brain, forming the gut-microbiota-brain axis. Obesity and high-fat diets can alter the gut microbiota, which in turn affects human emotions and behavior through this axis ^[30]^. Thirdly, obesity may trigger overactivity in the hypothalamic-pituitary-adrenal (HPA) axis, causing an excessive release of cortisol. Prolonged high levels of glucocorticoids can impair the function of the HPA axis, further contributing to the development and progression of depression ^[31]^. Fourthly, prolonged excessive energy intake can disrupt synaptic plasticity, leading to alterations in neuronal structure, reduced emotional processing capabilities, and behavioral abnormalities ^[32]^. For example, obesity can affect the synthesis, release, and transport of neurotransmitters like serotonin (5-HT) and dopamine, thereby influencing emotional regulation and reward mechanisms ^[33]^. Considering age-related physiological changes such as decreased antioxidant capacity, HPA axis dysfunction, and reduced gut microbiota diversity, middle-aged and older adults are more prone to these mechanisms, thereby increasing their risk of developing depression. Therefore, the association of obesity with depression was particularly pronounced in the middle-aged and elderly population, BRI, an obesity indicator that integrates height and WC, BRI can more accurately reflect the obesity status of this demographic and its relationship with the risk of depression.

27.	Kojta I, Chacinska M, Blachnio-Zabielska A. Obesity, bioactive lipids, and adipose tissue inflammation in insulin resistance. Nutrients (2020) 12(5). doi: 10.3390/nu12051305.PMID: 32375231

28.	Bhatt S, Nagappa AN, Patil CR. Role of oxidative stress in depression. Drug Discov Today (2020) 25(7):1270-76. doi: 10.1016/j.drudis.2020.05.001.PMID: 32404275

29.	Zhang W, Xiao D, Mao Q, Xia H. Role of neuroinflammation in neurodegeneration development. Signal Transduct Target Ther (2023) 8(1):267. doi: 10.1038/s41392-023-01486-5.PMID: 37433768

30.	Schachter J, Martel J, Lin CS, Chang CJ, Wu TR, Lu CC, et al. Effects of obesity on depression: a role for inflammation and the gut microbiota. Brain Behav Immun (2018) 69:1-08. doi: 10.1016/j.bbi.2017.08.026.PMID: 28888668

31.	Schonning T, Dahl HJ, Hummelen B, Ulberg R. Do sleep disturbances improve following psychoanalytic psychotherapy for adolescent depression? Int J Environ Res Public Health (2022) 19(3). doi: 10.3390/ijerph19031790.PMID: 35162813

32.	Jacques A, Chaaya N, Beecher K, Ali SA, Belmer A, Bartlett S. The impact of sugar consumption on stress driven, emotional and addictive behaviors. Neurosci Biobehav Rev (2019) 103:178-99. doi: 10.1016/j.neubiorev.2019.05.021.PMID: 31125634

33.	Jiang C, Zhan Q, Zeng C. The 5-ht-related gut-brain axis in obesity. Life Sci (2024) 358:123171. doi: 10.1016/j.lfs.2024.123171.PMID: 39447731

**5.4** Practical Applications: Discuss how BRI could be integrated into clinical practice or public health policies, including the feasibility of using the 4.29 threshold in depression screening programs.

**Author Response:**

Thank you for your valuable suggestions. In the Discussion section, we present reasonable measures to integrate BRI into clinical practice and public health policy, including the feasibility of applying the 4.29 threshold.details are as follows:(lines 321-336 on page 18).

In clinical settings, BRI can be employed as an extra tool to check for depression risk. Since it's easy to calculate, BRI works well in primary care places and health check-ups. Clinical practitioners can integrate BRI into routine health assessments for middle-aged and older adults, complementing these assessments with mental health scales such as CESD-10 for comprehensive screening. According to the established BRI threshold (4.29), the middle-aged and older adults can be categorized into two groups: low risk of depression (BRI < 4.29) and high risk of depression (BRI ≥ 4.29). For the low-risk group, routine health education and regular follow-up are sufficient. In contrast, for high-risk individuals, it is advisable to intensify monitoring of depressive symptoms and implement early intervention strategies, including psychological counseling and lifestyle recommendations. Furthermore, BRI can be used in conjunction with traditional indicators such as BMI and WC. In comparison to BMI, BRI more accurately reflects the distribution of abdominal fat, aiding in the identification of depression-prone individuals who are "normal weight but have abdominal obesity.

Thank you once again for your positive and constructive suggestions on our manuscript. We hope our responses and the revised manuscript are satisfactory. We are looking forward to having our work published. If you have any further questions or need any additional information, please feel free to contact us. We are more than happy to provide any necessary clarifications.

Best regards.

Yours sincerely,

Corresponding author:

Name: Hua Lin

E-mail: 1264615433@qq.com
